# Generational Differences in Dietary Behaviours: A Cross-Sectional Study of Generations X, Y, and Z

**DOI:** 10.12688/f1000research.167810.1

**Published:** 2025-09-02

**Authors:** Nasser S Alqahtani, Saja Alanazi, Reema Almuhayd, Ethar Alanazi, Jenan Alanazi

**Affiliations:** 1 Community Health Department, Northern Border University, Arar, Northern Borders Province, 91431, Saudi Arabia

**Keywords:** Generation X, Generation Y, Generation Z, dietary behaviour, Saudi Arabia, nutrition, public health, obesity

## Abstract

**Background:**

Dietary behaviours influence obesity and chronic disease. In Saudi Arabia, Westernised diets and sedentary lifestyles have driven rising obesity. This study explores generational dietary patterns to inform Saudi Vision 2030 planning.

**Methods:**

A cross-sectional study of 1,153 individuals was conducted in Saudi Arabia. These participants came from three generations: Generation X (born 1965–1980), Generation Y (born 1981–1996), and Generation Z (born 1997–2012). Participants were recruited via digital platforms (social media, email lists, and university networks). They also completed a validated, self-administered online questionnaire that captured 24-hour dietary recall, food-choice determinants, and relevant lifestyle factors. All data were statistically analysed using IBM SPSS Statistics version 25 and considered significant at p values < 0.05.

**Results:**

Generational differences were statistically significant (p<0.001). For Generation Z, 34.1% reported consuming soft drinks more than three times weekly, compared to 6.4% for Generation X and 20.8% for Generation Y. Generation Z also had the lowest intake of fruits (only 4.8% reported ≥3 servings per day) and vegetables (8.4% met ≥3 servings per day). These individuals were more influenced by peers, taste (60.6%), and price (10.5%) than by nutrition. Conversely, Generations X and Y prioritised long-term health (69.5% and 38.9%, respectively) and nutritional value (71.1% and 38.5%, respectively). Gen Z favoured restaurant dining (40.3%), showed higher peer influence (63.2%), and more frequent meal skipping (88.3%). Furthermore, favourable nutritional intake, including water and fruits/vegetables, declined across generations, with Gen Z consuming more soft drinks (59.2%) and snacks (51.6%) than Gen X.

**Conclusions:**

Generational dietary differences in Saudi Arabia reflect global and local shifts. Targeted public health strategies, digital interventions addressing affordability and appearance for Gen Z, and reinforced traditional education for Gen X/Y are essential. These insights support school and community nutrition policies aligned with Saudi Vision 2030.

## Introduction

Understanding dietary behaviours is a central concern in nutrition and public health. This is because eating habits are closely linked to the development and prevention of chronic diseases, obesity, and overall well-being. ^1^ Patterns such as food selection, meal timing, beverage consumption, and frequency of eating out significantly affect caloric intake, nutrient adequacy, and metabolic health. ^2^ Poor dietary habits include high intake of processed foods and sugary drinks and low consumption of fruits and vegetables. These habits are, in turn, strongly associated with non-communicable diseases, such as type 2 diabetes and cardiovascular disease. ^1, 3^

Globally, urbanisation, technological advancements, and cultural changes have driven significant shifts in dietary behaviour. ^4^ The spread of fast food, the convenience of food delivery services, and aggressive food marketing have led many young people to adopt Western-style diets that are high in calories and low in nutrients. ^5^ Increased screen time and digital engagement further promote food choices driven by trends, convenience, and taste over nutritional quality. ^6^ These global patterns are evident in Saudi Arabia, where modernisation and urban living have contributed to widespread dietary changes. The decline of traditional, home-prepared meals and the increased availability of energy-dense, processed foods have been linked to the nation’s rising obesity rates. ^1^ Indeed, recent findings underscore poor dietary patterns and physical inactivity as significant public health concerns. ^2^

Birth cohorts are the cohorts of individuals who were born during a specific date range and share similar cultural experiences. Eating is a kind of behaviour, and, like much other behaviour, is largely shaped by generational dynamics. International research has demonstrated that older adults are more inclined to value the quality of food, its health effects, and traditional dietary behaviours (e.g., eating homemade food and avoiding food waste). ^4, 5^ In contrast, younger generations usually prioritise ease and price, and are more affected by digital media. This means that they eat higher amounts of fast food, snacks, and sugary drinks than older generations. ^4, 5^ As mentioned, the younger generation in Saudi Arabia is showing a noticeable transition from traditional eating habits to Westernised dietary habits. Economic development factors, increasing global fast food chains, and social media influence have reshaped the traditional culture and resulted in a higher consumption of fast food, a lack of regular meal timing, and a sedentary lifestyle. ^7^ These changes are partly driving the country’s rising burden of diet-related, chronic diseases.

While there are clear trends, relatively little research has compared dietary behaviours between different generations in Saudi Arabia. Most of the current literature focuses specifically on the youth or some other population group. This means that little is known about how the motives and behaviours of one generation differ from another. ^3, 6^ Arguably, this lacuna is hampering the development of specific, age-oriented interventions in public health. Indeed, exploring generational differences in diet is key to developing targeted healthy eating campaigns that reflect the values, drivers, and trends that are specific to the different generations. This is in line with Saudi Vision 2030, which places an emphasis on health improvement and decreasing chronic diseases by living a healthy lifestyle. Thus, culturally informed knowledge about intergenerational dietary practices is useful for community health interventions and health policy at a national level.

Our primary aim in this study is to compare dietary patterns and influencing factors across Generations X, Y, and Z (Gen X, Gen Y, and Gen Z) in Saudi Arabia. Specifically, we seek to assess generational differences in food and beverage consumption frequencies (e.g., water, soft drinks, fruits, and vegetables) and explore the underlying motivations for healthy eating, including social influences. In doing so, our study addresses two main research questions:

1. Do significant differences exist in dietary behaviours among members of Gen X, Gen Y, and Gen Z in Saudi Arabia?
2. Are younger generations more influenced by social factors and less aligned with healthy eating practices than older generations?

## Methods

### Study design and setting

In this research, we employed a cross-sectional quantitative study design using a self-administered online survey. The choice of an online format facilitated wide geographic coverage across Saudi Arabia and preserved participant anonymity. The survey was accessible for a period of 10 days, beginning in April 2025. Recruitment and participation were conducted entirely through digital platforms to ensure national-level reach.

### Participants

Participants were eligible for inclusion if they were aged 18 years or older, in Saudi Arabia, and were able to answer the questionnaire. For the purposes of this research, Gen X includes those born between 1965 and 1980, Gen Y refers to those born between 1981 and 1996 (‘the Millennials’), and Gen Z are those born between 1997 and 2012. ^4^ Exclusion criteria included incomplete survey responses and participants whose ages did not fall within the generational categories of interest (i.e., outside the birth years for the three relevant generations). A total of 1,116 valid responses were collected and analysed. Of these, 622 participants (53.94%) were classified as Gen Z (born 1997–2012), 265 (22.98%) were classified as Gen Y (born 1981–1996), and 266 (23.07%) were classified as Gen X (born 1965–1980).

### Questionnaire design

The instrument consisted of 26 structured questions designed to capture a comprehensive overview of participants’ dietary behaviours and influences. A total of 21 items assessed 24-hour dietary recall questionnaire and habitual food consumption. One item classified respondent into generational cohorts based on age, while three questions gathered demographic information, including gender, education, and geographic region. One additional item was included in the pilot phase to evaluate clarity.

The questionnaire encompassed a range of domains relevant to understanding dietary behaviour across generations. Specifically, it assessed the frequency of meals and the intake of various beverages, particularly water and sugary drinks. It also evaluated the consumption patterns of major food groups, including seafood, meat, fruits, and vegetables. Beyond intake, the survey explored motivations for healthy eating and the criteria individuals consider when selecting foods, such as price, taste, and nutritional value. The questionnaire also examined the influence of peers on eating decisions and the prevalence of screen use during meals. These factors offered insights into both the psychological and behavioural phenomena shaping dietary choices. The survey was pilot tested for clarity and comprehensibility before full deployment. Its development was, in turn, informed by existing literature on generational differences in dietary behaviour. The questionnaire underwent pilot testing to evaluate clarity beside content validity and face validity. Internal consistency reliability was assessed, and the tool demonstrated acceptable reliability for the included dietary behaviour domains.

### Data collection

The survey link was disseminated via social media, email distribution lists, and academic institutional networks. Participants were informed that the survey would take approximately five to ten minutes to complete. All responses were anonymous, and participation was entirely voluntary. Implied informed consent was obtained when participants agreed to proceed after reading the study introduction.

### Data transformation and analysis

To facilitate an analysis of dietary patterns, we transformed several Likert-scale and categorical variables into binary indicators reflecting favourable versus unfavourable dietary behaviours based on established nutritional guidelines. This dichotomisation allowed for a clear interpretation of population-level dietary patterns while maintaining sufficient granularity for between-group comparisons. The code “1” represented the nutritionally favourable option, and “0” represented the unfavourable or suboptimal option. This was based on the following criteria:

*Healthy eating behaviours*

• Trying to eat healthily: Favourable (1) = “Yes” or “Sometimes”; Unfavourable (0) = “No”.
• Meal choice basis: Favourable (1) = health-motivated choices; Unfavourable (0) = choices based on taste or price.
• Beverage consumption: Soft drinks: Favourable (1) = none or ≤1/week; Unfavourable (0) = frequent intake (≥2–3/week).
• Water intake: Favourable (1) = ≥6 cups/day; Unfavourable (0) = <6 cups/day.

*Food group consumption*

• Fruits/vegetables: Favourable (1) = ≥1 serving/day; Unfavourable (0) = none or irregular intake.
• Dairy: Favourable (1) = any consumption; Unfavourable (0) = none.
• Meat/seafood: Favourable (1) = moderate intake (weekly but not daily); Unfavourable (0) = daily or no intake.
• Snacks/processed carbs: Favourable (1) = limited (≤1/day); Unfavourable (0) = frequent (≥2/day).

*Behavioural and contextual factors*

• Cooking methods: Favourable (1) = steaming/grilling; Unfavourable (0) = frying/mixed cooking.
• Meal skipping: Favourable (1) = no skipping; Unfavourable (0) = skipping meals.
• Mindful eating: Favourable (1) = no screen use during meals; Unfavourable (0) = distracted eating.

Meal size (small, medium or large) and usual place the meal is taken in (Home, restaurant, desert safari, Etc) were also recorded.

### Statistical analysis

Descriptive statistics compared dietary behaviours across generations (X, Y, and Z). Continuous variables (e.g., meal count per day) were analysed using Kruskal-Wallis, while binary/categorical variables were compared using Pearson’s χ ^2^ tests. We then generated two tables: one retaining original response scales and another using binary classification to highlight favourable/unfavourable patterns. All analyses were conducted in IBM SPSS Statistics version 25, with statistical significance set at P < 0.05.

## Results

### Sample characteristics

**Image 1.**
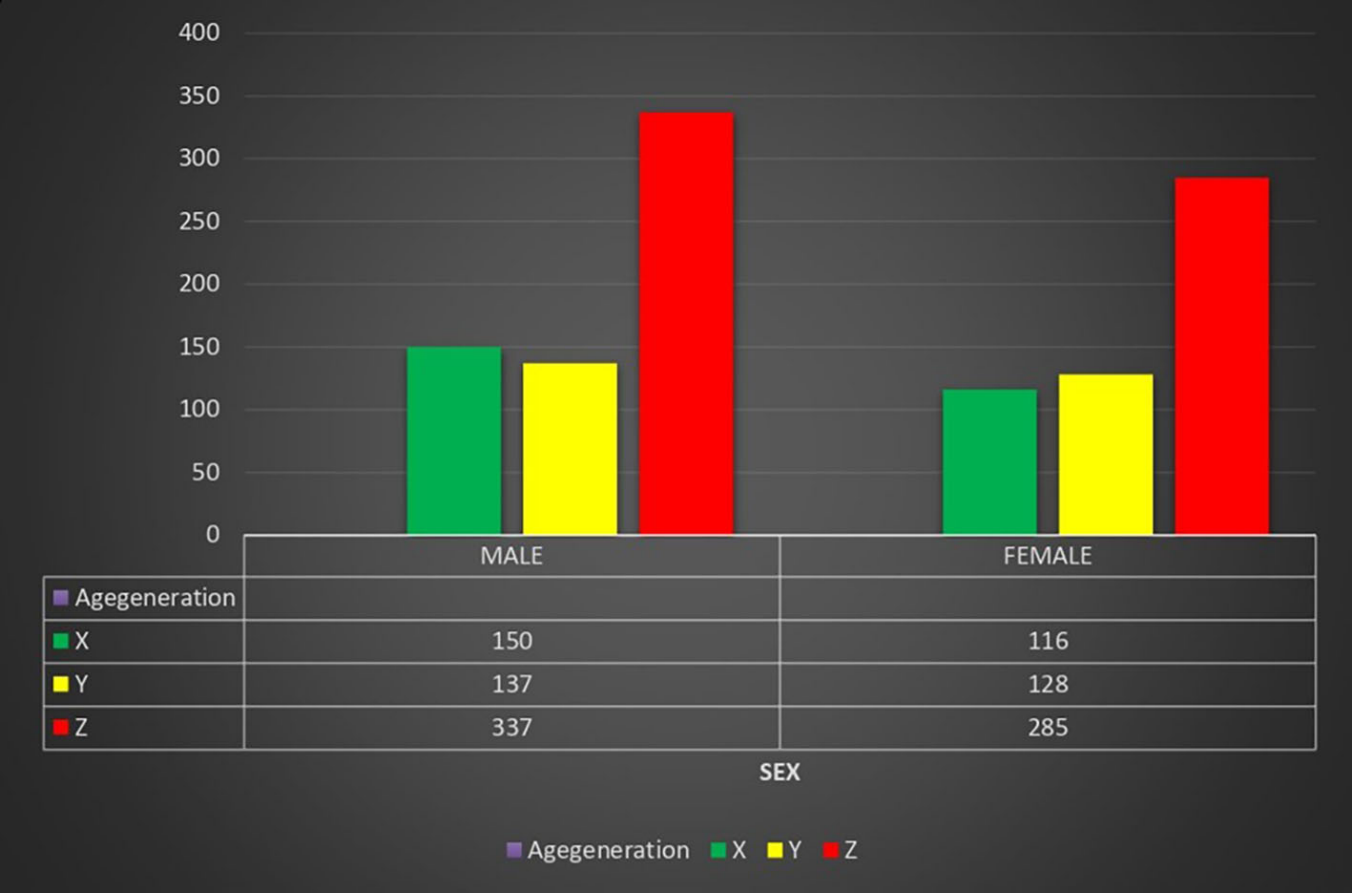
Distribution of generation on the basis of sex.

Image 1 shows a nearly balanced distribution across genders and age groups. Among the total 1,153 participants, 624 (54.1%) were male and 529 (45.9%) were female. In Generation X, males slightly outnumbered females; in Generation Y, females slightly exceeded males; and in Generation Z, males were more than females. Overall, Generation Z had the highest representation across both sexes, making up more than half of the total sample (53.9%).

**Image 2.**
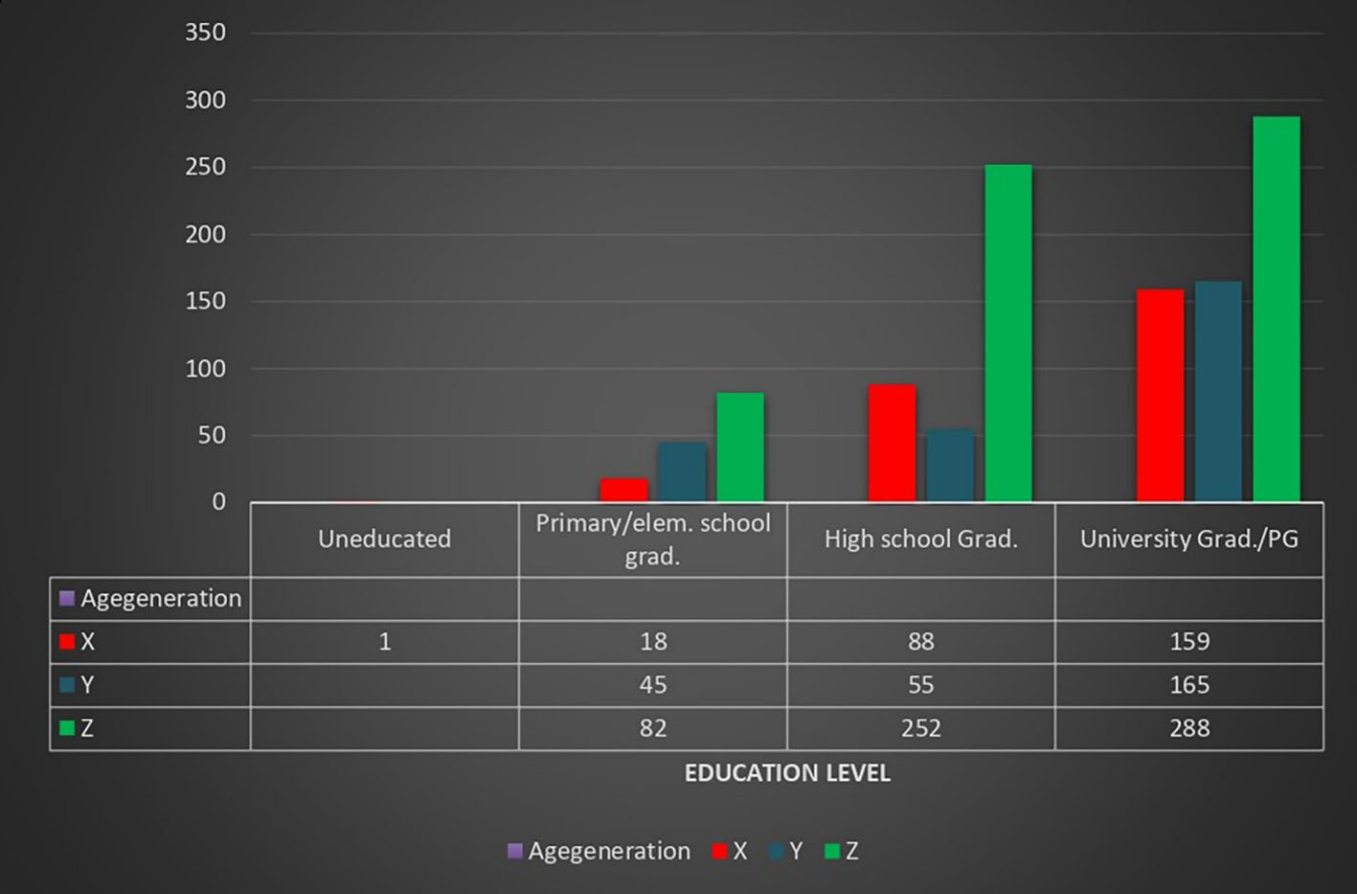
Distribution of generation on the basis of education level.

Image 2 shows the majority of participants across all generations were university graduates or postgraduates, highest in Gen-Y (62.3%) and Gen-X (59.8%), followed by Gen-Z (46.3%). High school graduates were more prevalent in Gen-Z (40.5%) compared to Gen-X (33.1%) and Gen-Y (20.8%). Primary school graduates were relatively few, most common in Gen-Z (13.2%). Only one participant (Gen-X) was uneducated. Overall, education levels were highest in older generations, with a notable portion of Gen-Z still at the high school level.

### Dietary pattern differences

The Table 1 reveals significant generational differences in dietary behaviors. Gen-X (n = 266) is the most health-conscious, with 73.7% trying to eat healthy, 71.1% choosing meals based on health benefits, and 88% preferring to eat with family. In contrast, Gen-Z (n = 622) is more influenced by peers (60.6%), taste (60.6%), and technology 73.5% use phones/tablets while eating, and 54.7% skip breakfast. Gen-Y (n = 265) shows intermediate patterns, with 58.5% trying to eat healthy and 44.2% motivated by weight control. Home-cooked meals are preferred across all groups, but Gen-Z shows the highest delivery use (16.7%). These numbers highlight a clear generational shift in food choices, motivations, and mealtime behaviors.

**Table 1.** Dietary behaviors and meal preferences across generations (Gen-X, Gen-Y, and Gen-Z).

Question	Response	Gen-X (n = 266)	Gen-Y (n = 265)	Gen-Z (n = 622)	P-value (χ ^2^)
Friend/colleagues influence to choose food	No (1)	162 (60.9%)	73 (27.5%)	229 (36.8%)	0.000 (84.29)
	Yes sometimes (2)	95 (35.7%)	168 (63.4%)	377 (60.6%)	
	Yes always (3)	9 (3.4%)	24 (9.1%)	16 (2.6%)	
Are trying to eat healthy	Yes (1)	196 (73.7%)	155 (58.5%)	202 (32.5%)	0.000 (159.65)
	No (2)	12 (4.5%)	14 (5.3%)	133 (21.4%)	
	Sometimes (3)	58 (21.8%)	96 (36.2%)	287 (46.1%)	
How do you choose meals	Colour/Taste (1)	65 (24.4%)	129 (48.7%)	377 (60.6%)	0.000 (141.13)
	Price (2)	12 (4.5%)	34 (12.8%)	65 (10.5%)	
	Health Benefits (3)	189 (71.1%)	102 (38.5%)	180 (28.9%)	
Motivation to consume healthy	Health/Prevention (1)	185 (69.5%)	103 (38.9%)	231 (37.1%)	0.000 (135.39)
	Control weight (2)	62 (23.3%)	117 (44.2%)	284 (45.7%)	
	Energy (3)	0 (0.0%)	11 (4.2%)	80 (12.9%)	
	Doctor advised (4)	19 (7.1%)	34 (12.8%)	27 (4.3%)	
Where do you prefer to eat	Home-cooked (1)	226 (85.0%)	201 (75.8%)	487 (78.3%)	0.000 (91.74)
	Delivery (2)	10 (3.8%)	20 (7.5%)	104 (16.7%)	
	Dine-in (3)	20 (7.5%)	33 (12.5%)	147 (23.6%)	
	Outdoors (4)	10 (3.8%)	11 (4.2%)	23 (3.7%)	
Cooking method you like	Mixed (1)	9 (3.4%)	5 (1.9%)	36 (5.8%)	0.000 (47.47)
	Plain (2)	189 (71.1%)	123 (46.4%)	365 (58.7%)	
	Barbeque (3)	65 (24.4%)	131 (49.4%)	206 (33.1%)	
	Steamed (4)	3 (1.1%)	6 (2.3%)	15 (2.4%)	
How you prefer your meal	Alone (1)	15 (5.6%)	36 (13.6%)	198 (31.8%)	0.000 (190.11)
	With family (2)	234 (88.0%)	201 (75.8%)	268 (43.1%)	
	With friends/colleagues (3)	17 (6.4%)	28 (10.6%)	156 (25.1%)	
What meal you skip	Breakfast (1)	51 (19.2%)	91 (34.3%)	340 (54.7%)	0.000 (195.21)
	Lunch (2)	35 (13.2%)	61 (23.0%)	127 (20.4%)	
	Dinner (3)	51 (19.2%)	61 (23.0%)	82 (13.2%)	
	Don’t skip (4)	129 (48.5%)	52 (19.6%)	73 (11.7%)	
Why you eat	When hungry (1)	241 (90.6%)	224 (84.5%)	508 (81.7%)	0.000 (30.11)
	Angry/Nervous (2)	17 (6.4%)	6 (2.3%)	23 (3.7%)	
	Bored (3)	8 (3.0%)	35 (13.2%)	91 (14.6%)	
While eating	Watching TV (1)	37 (13.9%)	119 (44.9%)	63 (10.1%)	0.000 (505.63)
	On phone/tablet (2)	35 (13.2%)	73 (27.6%)	457 (73.5%)	
	Playing games (3)	3 (1.1%)	96 (36.2%)	12 (1.9%)	
	Not watching/playing (4)	191 (71.8%)	64 (24.2%)	90 (14.5%)	

### Food preference differences

The Table 2, highlights significant differences (p < 0.001, except starch/carbs p = 0.004) in food preferences and eating habits across generations. Gen-X (n = 266) tends to consume fewer soft drinks (63.5% don’t drink), drinks more water (49.2% drink >10 cups/day), eats 3 meals daily (63.2%), and consumes more fruits (44% eat ≥3 servings) and vegetables (47.7% eat ≥3 servings). They prefer smaller restaurant meal sizes (59%). Gen-Z (n = 622) consumes soft drinks more frequently (34.1% >3 times weekly), drinks less water (69% drink ≤5 cups/day), eats fewer meals (only 26.2% eat 3 meals), consumes less fruit (44.5% don’t eat fruits) and vegetables (30.2% don’t eat vegetables), and prefers medium to large restaurant meal sizes (82%). Gen-Y (n = 265) shows intermediate patterns but consumes more meat weekly (36.6% one serve/week) and snacks mostly once daily (59.6%). Snack frequency is highest in Gen-Z, with 27.5% snacking 3+ times daily. Dairy consumption is highest in Gen-X (46.6% drink 3+ cups daily), while Gen-Z has the largest proportion (64.5%) consuming only one cup daily. Overall, these results indicate younger generations have less healthy eating habits compared to older generations.

**Table 2.** Food preference and eating habits across Generations (Gen-X, Gen-Y, and Gen-Z).

Question	Response	Gen-X (n = 266)	Gen-Y (n = 265)	Gen-Z (n = 622)	P-value (kruskal-wallis)
Soft drinks:	1 = once a week	51 (19.2%)	86 (32.5%)	137 (22.0%)	0.000 (79.76)
	2 = 2 times/3 time a week	29 (10.9%)	54 (20.4%)	156 (25.1%)	
	3 = more than 3 times weekly	17 (6.4%)	55 (20.8%)	212 (34.1%)	
	4 = no I don’t drink	169 (63.5%)	70 (26.4%)	117 (18.8%)	
Water drink:	1 = 5 cups or less per day	62 (23.3%)	119 (44.9%)	429 (69.0%)	0.000 (219.37)
	2 = 6 to 10 cups per day	73 (27.4%)	68 (25.7%)	148 (23.8%)	
	3 = more than 10 cups per day	131 (49.2%)	78 (29.4%)	45 (7.2%)	
Number of meals per day:	1 = 1 meal	7 (2.6%)	19 (7.2%)	145 (23.3%)	0.000 (113.60)
	2 = 2 meals	88 (33.1%)	100 (37.7%)	286 (46.0%)	
	3 = 3 meals	168 (63.2%)	131 (49.4%)	163 (26.2%)	
	4 = more than 3 meals	3 (1.1%)	15 (5.7%)	28 (4.5%)	
Fruit per day:	1 = I don’t eat fruits	31 (11.7%)	69 (26.0%)	277 (44.5%)	0.000 (194.92)
	2 = one serve fruit a day	83 (31.2%)	92 (34.7%)	234 (37.6%)	
	3 = 2 serve fruits a day	35 (13.2%)	92 (34.7%)	81 (13.0%)	
	4 = 3 serves fruits or more	117 (44.0%)	12 (4.5%)	30 (4.8%)	
Vegetable per day:	1 = I don’t eat vegetables	11 (4.1%)	27 (10.2%)	188 (30.2%)	0.000 (170.45)
	2 = one serve vegetables a day	92 (34.6%)	124 (46.8%)	260 (41.8%)	
	3 = 2 serve vegetables a day	36 (13.5%)	46 (17.4%)	122 (19.6%)	
	4 = 3 serves vegetables or more	127 (47.7%)	68 (25.7%)	52 (8.4%)	
Dairies consume:	1 = one cup daily	98 (36.8%)	101 (38.1%)	401 (64.5%)	0.000 (78.11)
	2 = 2 cups daily	33 (12.4%)	80 (30.2%)	105 (16.9%)	
	3 = 3 cups or more daily	124 (46.6%)	62 (23.4%)	57 (9.2%)	
	4 = I don’t consume any	11 (4.1%)	22 (8.3%)	59 (9.5%)	
Meat consumption:	1 = one or more serve daily	54 (20.3%)	94 (35.5%)	429 (69.0%)	0.000 (37.53)
	2 = one serve per week	47 (17.7%)	97 (36.6%)	269 (43.2%)	
	3 = 2 or 3 serve per week	145 (54.5%)	51 (19.2%)	314 (50.5%)	
	4 = 4 serve or more per week	20 (7.5%)	23 (8.7%)	141 (22.7%)	
Number snack per day:	1 = none	48 (18.0%)	26 (9.8%)	58 (9.3%)	0.000 (135.96)
	2 = once a day	189 (71.1%)	158 (59.6%)	243 (39.1%)	
	3 = twice a day	25 (9.4%)	62 (23.4%)	150 (24.1%)	
	4 = 3 times or more per day	4 (1.5%)	19 (7.2%)	171 (27.5%)	
Starch/carbs per day:	1 = none	11 (4.1%)	6 (2.3%)	25 (4.0%)	0.004 (11.07)
	2 = one serve per day	51 (19.2%)	55 (20.8%)	181 (29.1%)	
	3 = 2 serve per day	163 (61.3%)	109 (41.1%)	224 (36.0%)	
	4 = 3 to 4 serves per day	39 (14.7%)	85 (32.1%)	161 (25.9%)	
Seafood consumption:	1 = I don’t eat seafood	37 (13.9%)	51 (19.2%)	215 (34.6%)	0.000 (131.87)
	2 = one serve per day	14 (5.3%)	36 (13.6%)	31 (5.0%)	
	3 = once a week	108 (40.6%)	155 (58.5%)	348 (55.9%)	
	4 = 3 serve per week	107 (40.2%)	23 (8.7%)	20 (3.2%)	
Meal size (restaurants):	1 = small size	157 (59.0%)	85 (32.1%)	112 (18.0%)	0.000 (146.82)
	2 = medium size	89 (33.5%)	162 (61.1%)	369 (59.3%)	
	3 = big size	20 (7.5%)	18 (6.8%)	141 (22.7%)	

The Table 2, highlights significant differences (p < 0.001, except starch/carbs p = 0.004) in food preferences and eating habits across generations. Gen-X (n = 266) tends to consume fewer soft drinks (63.5% don’t drink), drinks more water (49.2% drink >10 cups/day), eats 3 meals daily (63.2%), and consumes more fruits (44% eat ≥3 servings) and vegetables (47.7% eat ≥3 servings). They prefer smaller restaurant meal sizes (59%). Gen-Z (n = 622) consumes soft drinks more frequently (34.1% >3 times weekly), drinks less water (69% drink ≤5 cups/day), eats fewer meals (only 26.2% eat 3 meals), consumes less fruit (44.5% don’t eat fruits) and vegetables (30.2% don’t eat vegetables), and prefers medium to large restaurant meal sizes (82%). Gen-Y (n = 265) shows intermediate patterns but consumes more meat weekly (36.6% one serve/week) and snacks mostly once daily (59.6%). Snack frequency is highest in Gen-Z, with 27.5% snacking 3+ times daily. Dairy consumption is highest in Gen-X (46.6% drink 3+ cups daily), while Gen-Z has the largest proportion (64.5%) consuming only one cup daily. Overall, these results indicate younger generations have less healthy eating habits compared to older generations.

**Image 3.**
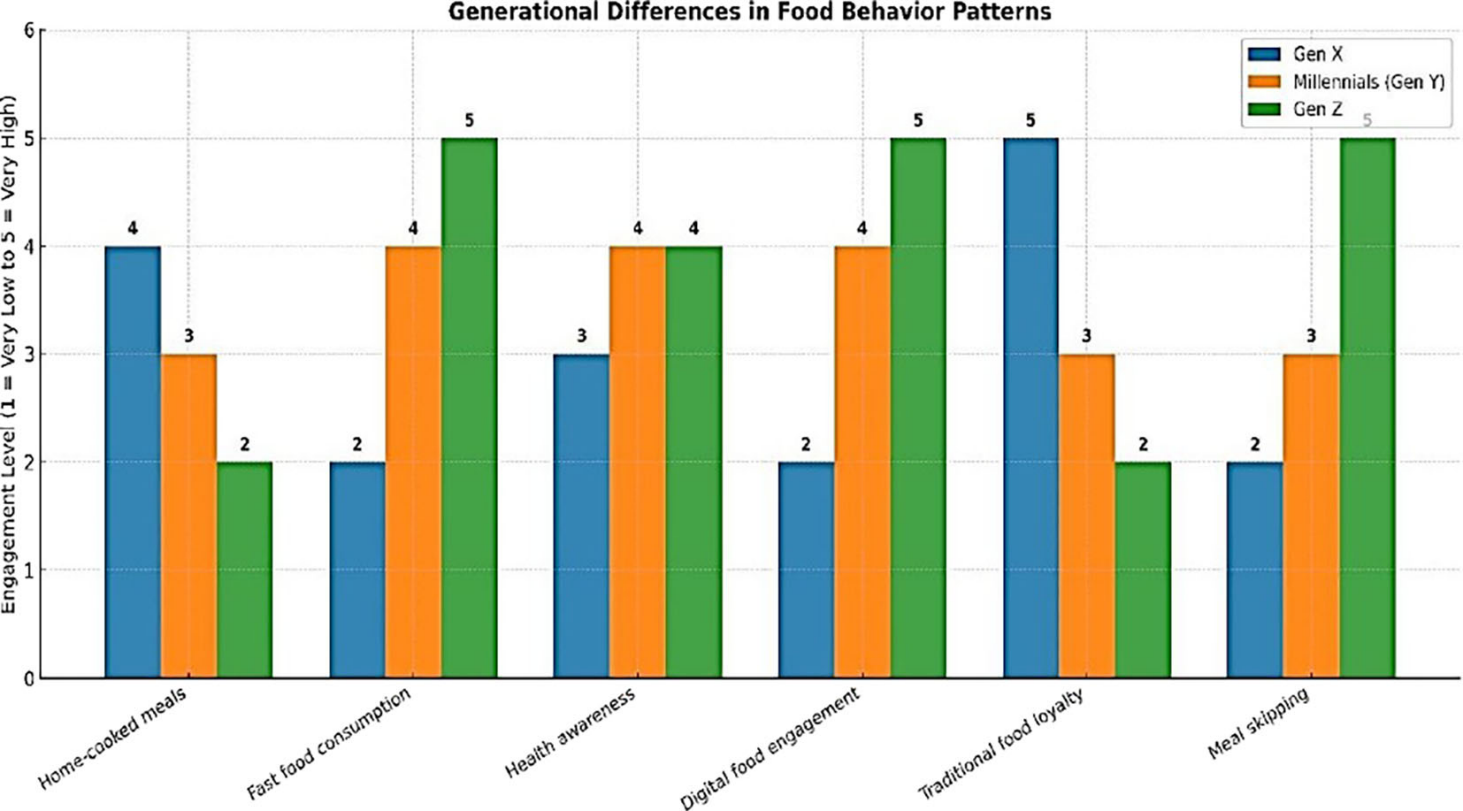
Eating Habits pattern among Generation X, Y AND Z.

Image 3, demonstrate Gen X shows strong loyalty to traditional food, high preference for home-cooked meals, and rarely skips meals. Millennial (Gen Y) display moderate home-cooking habits, high fast food intake, strong health awareness, and frequent digital engagement with food content. Gen Z leads in fast food consumption and digital food engagement is trend-driven in health awareness, weak in traditional food loyalty, and most likely to skip meals. Overall, dietary behavior shifts toward convenience and digital influence in younger generations.

## Discussion

This study identified statistically significant generational differences in dietary behaviours among Saudi adults (p < 0.001). Gen Z demonstrated the highest consumption of sugary drinks and the lowest intake of fruits, vegetables, and seafood. This imbalance reflects global concerns over the nutritional quality of modern diets among the youth. ^8^ Notably, when dietary behaviours were compared to standard nutritional recommendations, such as consuming at least two to three servings of fruits and three or more servings of vegetables per day, only Gen X showed relatively adequate intake levels. Over 44% of Gen X met the fruit intake recommendation, while only 4.8% of Gen Z and 4.5% of Gen Y reached the threshold of three or more daily servings. Similarly, 47.7% of Gen X met the vegetable guideline compared to just 8.4% for Gen Z. These patterns highlight substantial gaps in dietary quality among younger generations, particularly Gen Zers, whose diets fall well short of national and international nutritional guidelines. In contrast, Gen X and Y reported more balanced dietary patterns, with a stronger emphasis on healthier food choices. Motivational drivers also differed: Long-term health considerations primarily motivated Gen X and Y, while Gen Z was more motivated by weight concerns and body image (which might limit their fitness orientation to appearance rather than well-being). ^9^ Peer influence had a much greater influence in Gen Z, which emphasises the salient influence of social and digital dieting norms. Food selection criteria also varied between generations, with Gen X and Y being more focused on nutritional value, and Gen Z more concerned with taste, price, and convenience. ^10^

This study’s results align with international literature that emphasises generational differences in food-related attitudes and behaviour. In Poland, when comparing generations, Gen Xers place greater emphasis on the quality of food and its nutritional value, while Gen Zers are more interested in price and convenience and less interested in eco- or health-aware consumption. ^5^ Similar studies in Turkey observed that Gen Zers are influenced by social media and food trends when making dietary choices, while Gen Xers are more health conscious (despite limited nutritional knowledge). ^4^ These patterns align with, and are corroborated by, broader evidence that younger age groups appear to consistently consume more processed, animal-based, and convenience foods, while older age groups show a higher attachment to plant-based, traditional, and healthy foods. ^10, 11^ The similarities in trends across regions suggest that intergenerational dietary changes are driven by global technological, economic, and social changes. Our study results confirm the above, with Gen Z’s diet resembling other young groups around the world. This diet is, notably, low in nutritional quality and highly driven by social factors. This is indicative of both local issues and global dietary patterns. It also highlights the importance of culturally appropriate generation-specific nutrition policies and interventions.

The differentiation across generations in Saudi Arabia is also influenced by a rapid transition of the country’s socio-economic status and food environment. Indeed, most local studies have reported a high consumption of fast food and sugary drinks, especially among the youth, which leads to an increase in rates of obesity and non-communicable diseases. ^1, 3, 12^ Thus, our study’s findings (vis-à-vis generational differences) can be explained in terms of lifestyle frameworks and behavioural motivation. Gen Z dietary practices seem to be influenced by greater time spent engaging with digital platforms, regular exposure to social media, and a vulnerability to marketing trends. These factors lead to their high intake of sugary beverages and fast foods, and a preference for taste and cost over nutritional value. ^4, 8^ The current generation’s food preferences are, in turn, a manifestation of an increasing general trend toward convenience-oriented eating, and away from home-made and traditional eating. As intimated, this shift is frequently based on peer norms and/or trend-related consumption. ^10^ In contrast, Gen X and Y seem to be more encouraged by prevailing health goals and traditional food values, which can be attributed to life stage maturity, awareness of chronic diseases, and family responsibilities. These factors encourage older individuals to maintain more balanced dietary behaviours. ^9^

Generational distinctions in motivation are also revealing. It is possible that Gen Z’s increased emphasis on weight control, rather than overall health or disease prevention, reflects an increasing emphasis on body image and outward appearance. This could be related to the influence of the ‘perfect image’ or weight-related messages on social media that encourage dieting without adequate nutrition. ^8^ Although such motivations can promote dietary self-regulation, they can also cultivate disordered eating patterns and stress-induced eating behaviours. This can, in turn, wreak havoc on people’s physical and mental well-being. ^13^ In contrast, Gen X’s and Y’s focus on nutrition and health maintenance points to food choices being largely made in preventative and wellness terms.

When it comes to meal frequency, Gen Z’s lower reported rates might be correlated to sporadic eating patterns related to time, changing lifestyle rhythms, and/or attempts to adhere to fad diets (e.g., intermittent fasting). These habits are consistent with the results of global and local studies that have reported how many young adults occasionally miss meals, consume more snacks, and/or replace structured meals with more convenient, but less nutritious, options. ^12, 14^ These behaviours highlight the need for managing not only what young people eat but also how and when they eat in a wider psychosocial and cultural context. Thus, future interventions should take into account a combination of convenience, social influence, and mental health when trying to engender healthier lifestyles among the youth.

This shift across generations implies that age-targeted public health strategies, which reflect each generation’s unique drivers and food attitudes, are now more critical than ever. When it comes to Gen Z, the most effective approaches will resonate with what is important to members of that group in terms of affordability, aesthetics, convenience, and digital connectivity. Messaging that links healthy eating to body image, performance, and social relevance especially when delivered via influencers, short-form videos, or interactive apps—may be more persuasive than traditional health education approaches. ^6, 8^ Gen X and Y are, conversely, already more closely aligned with healthy habits and long-term health practices. Reinforcing knowledge through lifestyle-based campaigns and community programs can, thus, help these older generations sustain and enhance their healthy habits. ^9^

The notion of tailoring interventions to generations is consistent with the public health objectives of Saudi Vision 2030 a blueprint that aims to decrease the national burden of chronic diseases through lifestyle reformation. In a nation with increasing rates of obesity, diabetes, and cardiovascular disease particularly among the youth policy interventions should be responsive to the different drivers and media types each generation consumes. ^15, 16^ There might not be a one-size-fits-all solution, and generationally informed targeted messages and campaigns may be most effective in driving meaningful dietary improvements and sustained behaviour change.

It follows that technology should be foundational in this work, especially when it comes to reaching Gen Z. Tools such as gamified nutrition apps, personalised meal plans, or social media challenges that support good health can drive engagement and measure accountability among this techno-empowered generation. Indeed, culturally tailored digital interventions have been reported to enhance health literacy and behaviour change among Saudi youth. ^6^ For the older generation, wearable devices that provide reminders, suggest recipes, or monitor clinical objectives could help maintain good habits in a friendly and supportive manner. Using these tools in a culture-appropriate context has great potential to make a scalable and sustainable impact.

Our study has several key strengths. It is the first of its kind in Saudi Arabia to investigate generational disparities in dietary practices. In doing so, we provided locally adapted evidence pertinent to age-related differences in food choices, motivations, and lifestyle variables. Moreover, the large sample of 1,116 respondents across Gen X, Y and Z increases the generalizability of our results and the ability to conduct sensitive comparisons. Our validated literature-based questionnaire covered 24-hour dietary recall, consumption behaviours, and motivational/psychosocial determinants. This guaranteed the gathered information’s robustness and comprehensiveness. Such methodological merits render this study a significant contribution to the project of exploring age-specific dietary patterns in the Kingdom.

However, several limitations must be acknowledged. The study’s cross-sectional nature restricts causal inference, making it difficult to determine whether generational identity directly influences dietary patterns or if there are other confounding variables at play. As with many self-administered surveys, responses are subject to recall inaccuracies and social desirability bias, especially when it comes to sensitive or socially influenced behaviours (e.g., fast food consumption and dieting motives). Although reflective of the digital data collection method, the overrepresentation of Gen Z participants could, in turn, skew intergenerational comparisons. The absence of biochemical or clinical markers also limits the ability to correlate reported dietary behaviours with actual health outcomes (a gap that has also been noted in previous national surveys). ^16, 17^

To build on these findings, future researchers should adopt longitudinal approaches to explore how dietary behaviours evolve across the life course and respond to changing social or economic conditions. Moreover, incorporating qualitative components, such as interviews with participants from each generation, would uncover deeper motivational drivers and cultural narratives that shape eating patterns (as seen in recent multi-regional and migration studies. Doing so could aid explorations of the relative pervasiveness versus cultural specificity of generation-based trends. Finally, the application of serum-based nutritional assessments and clinical measures would facilitate deeper insights into the health consequences of the relevant generational dietary divergences and justify evidence-based public health strategies.

## Conclusions

This study substantiates how intra-generational variations in dietary practice are distinguishable and significant in Saudi society. Young generations, including Gen Z, exhibit an eating profile characterised by the increased consumption of sugary drinks and low intake of fruits,  vegetables, or sea food. These individuals are more motivated by aesthetics and peer influence than by long-term health concerns. Conversely, older generations are more health-conscious and tend to focus on nutrition quality when making food choices.

These findings underscore the urgent need to implement generation-specific public health interventions and nutritional education campaigns tailored to each cohort’s unique preferences, influences, and challenges. Digital health interventions, such as gamified nutrition apps and social media based outreach, should be leveraged to engage Gen Z and promote healthier eating behaviours among that group’s members. These kinds of targeted strategies are essential for effectively promoting healthier eating habits and reducing the burden of diet-related chronic diseases across the Saudi population.

## Preregistered data analysis

The authors did not pre-register the research at an independent registry.

## Ethical considerations

The study was conducted in accordance with ethical research standards. Ethical approval was sought from the appropriate institutional review board [NBU/2025/054], and participants provided informed consent through their participation. No personally identifiable data was collected, which ensured confidentiality and data privacy.

Ethics approval: Northern Border University; written informed consent was implied by survey participation.

## Data Availability

All data underlying the results of this study are openly available in the Zenodo repository under a CC-BY 4.0 licence. The dataset includes anonymized raw data from the online questionnaire, the values underlying all reported means, standard deviations, tables, and figures, as well as the points extracted from images for analysis. The data can be accessed via DOI: 10.5281/zenodo.16673563. ^18^ No embargoes or access restrictions apply. Data are available under the terms of the Creative Commons Attribution International license (CC BY 4.0).

## Software availability

No new software was developed for this study. Data analysis was performed using IBM SPSS Statistics version 25, which is commercially available. No custom scripts or code were generated.
