# Supplementary material for: Generational Differences in Dietary Behaviours: A Cross-Sectional Study of Generations X, Y, and Z
Source: F1000Res. 2025 Sep 2;14:858. [Version 1] doi: 10.12688/f1000research.167810.1 (PMC12455175; doi:10.12688/f1000research.167810.1)
Supplement: Peer review history

# Reviewer report: Reviewer response for version 1

**DOI:** 10.5256/f1000research.184953.r412978

**Published:** 2025-09-22

**Authors:** Saeed Soliman

**Affiliations:** 1 Cairo University, Cairo, Egypt

**Overall recommendation**

 Accept.

 The manuscript addresses a timely public-health question with a large national sample and provides actionable, generation-specific insights aligned with Saudi Vision 2030. The study is clearly written, methods are appropriate for the research questions, and data/ethics statements are transparent.

**Significance & originality**

 The paper fills a documented gap by comparing Generations X, Y, and Z within one Saudi dataset, linking behavioral patterns to practical policy levers (digital engagement for Gen Z; reinforcement strategies for Gen X/Y). The results are consistent with international trends while offering culturally specific nuances, enhancing relevance for regional policy and program design.

**Methodological soundness**

 The cross-sectional online survey, predefined generational bands, and use of χ²/Kruskal–Wallis tests are appropriate. The transformation of variables into favorable/unfavorable indicators aids interpretability for public-health stakeholders. The large sample (N>1,100) enables stable between-group comparisons.

**Interpretation & presentation**

 Findings are presented clearly, with coherent narrative links from statistics to policy implications (e.g., peer/taste/price salience in Gen Z vs health/nutrition salience in older cohorts). Conclusions are proportionate to the design and avoid causal claims.

**Minor suggestions (non-blocking)**

  

- Clarify measurement details: define “serving” for fruits/vegetables; justify the ≥6 cups/day water threshold; specify whether the “24-hour recall” was single-day and how habitual intake was inferred.
- Questionnaire validity: you note acceptable internal consistency—please provide the specific reliability indices (e.g., Cronbach’s α) for relevant domains.
- Sampling/generalisability: briefly acknowledge potential selection bias from digital recruitment and the over-representation of Gen Z; if feasible, add a sensitivity check (e.g., age- or sex-weighted descriptives) in a supplement.
- Tables/figures polish: ensure figure alt text/captions are self-contained; consider vector formats over GIFs; remove the duplicated paragraph describing Table 2.

Is the work clearly and accurately presented and does it cite the current literature?

Yes

If applicable, is the statistical analysis and its interpretation appropriate?

Yes

Are all the source data underlying the results available to ensure full reproducibility?

Yes

Is the study design appropriate and is the work technically sound?

Yes

Are the conclusions drawn adequately supported by the results?

Yes

Are sufficient details of methods and analysis provided to allow replication by others?

Yes

Reviewer Expertise:

Public health, primary care, family practice, T2DM HTN

I confirm that I have read this submission and believe that I have an appropriate level of expertise to confirm that it is of an acceptable scientific standard.

# Reviewer report: Reviewer response for version 1

**DOI:** 10.5256/f1000research.184953.r412981

**Published:** 2025-09-17

**Authors:** Nawaf Alruwaili

**Affiliations:** 1 King Saud University, Riyadh, Saudi Arabia

This article is of significance because it addresses an important research question with a clear and technically sound design. The study contributes to advancing knowledge in its field, particularly by providing methodologically rigorous data and drawing conclusions that are supported by the results. The clarity of presentation and relevance to current scientific debates also enhance its impact. Furthermore, the availability of data and reproducibility of methods strengthen its value as a reference for future work. The overall contribution is meaningful and has the potential to influence both future research directions and practical applications.

Is the work clearly and accurately presented and does it cite the current literature?

Yes

If applicable, is the statistical analysis and its interpretation appropriate?

Yes

Are all the source data underlying the results available to ensure full reproducibility?

Yes

Is the study design appropriate and is the work technically sound?

Yes

Are the conclusions drawn adequately supported by the results?

Yes

Are sufficient details of methods and analysis provided to allow replication by others?

Yes

Reviewer Expertise:

Nutrition Epigenetics, Nutrition-Nanotechnology, Micronutrients Deficiencies, Nutrition Support, Nutritional Risk Screening and Assessment, Public Health Nutrition, Nutrition Policy and Promotion, Nutrition and Health Issues

I confirm that I have read this submission and believe that I have an appropriate level of expertise to confirm that it is of an acceptable scientific standard.
